# Assessment of the Quality of Life of Vitiligo Patients: A Cross-Sectional Study in the Eastern Region of Saudi Arabia

**DOI:** 10.7759/cureus.65873

**Published:** 2024-07-31

**Authors:** Aminah A Alhumam, Ghadeer A Alibraheem, Heba Y Alojail, Ali A Al Ibraheem

**Affiliations:** 1 Dermatology, King Faisal University, Al Ahsa, SAU; 2 Medicine, King Faisal University, Al Ahsa, SAU

**Keywords:** vitiligo quality of life instrument, dermatology life quality index, quality of life, eastern region of saudi arabia, vitiligo

## Abstract

Background

Vitiligo is a prevalent skin disease that results from the loss of melanocytes and subsequent hypo-melanosis, resulting in the depigmentation of the skin. It not only presents as pathological manifestations but also imposes a substantial psychological burden and exerts a significant influence on the quality of life (QOL) of individuals. This research proposal seeks to systematically explore the association between vitiligo and the QOL of affected individuals, employing rigorous scientific methodologies to identify effective interventions aimed at improving their holistic well-being.

Methodology

It was a cross-sectional survey conducted in the Eastern Region of the Kingdom of Saudi Arabia (KSA). Data collection utilizes an online survey through Google Forms and employs the Dermatology Life Quality Index (DLQI). Appropriate statistical analyses were performed.

Results

Our study comprised 263 vitiligo patients, 55.1% of whom were females and 54.4% of whom were aged 18-30. Impact assessment revealed a substantial emotional toll (56.3% embarrassed), affecting daily activities (42.6%) and clothing choices (43.7%). Notably, 36.5% reported very much impact on relationships and 35.7% on sexual problems. Notably, 41.4% face a very large impact, and 35.4% face an extremely large impact. Linear regression identified a significant gender difference (p = 0.008), with males experiencing less QOL impact or females experiencing more QOL impact due to vitiligo. Age and marital status showed nonsignificant associations.

Conclusions

Our study highlights the substantial impact of vitiligo on the QOL among Saudi adults. Gender significantly influences severity, with females experiencing a more severe impact on the QoL, emphasizing the need for tailored interventions and support.

## Introduction

Vitiligo is the most prevalent among depigmenting disorders, which affects about 0.1%-2% of people worldwide [1]. The majority of studies show that females have a marginally higher citation. A characteristic of vitiligo is a long-standing acquired, idiopathic, progressive hypo-melanosis of the skin and hair with a complete lack of melanocytes under a microscope [2]. The face, lips, hands, feet, and genitalia are the skin regions that are most frequently impacted [3].

Determining the precise etiology of vitiligo is challenging, as it is thought to have a multifactorial origin. Factors such as genetics, oxidative stress, autoimmunity, neurological influences, toxic metabolites, and melanocyte growth deficiencies have all been proposed as potential contributors to the development of this condition [4].

Vitiligo is a significant factor in the lives of affected people, their families, and their social networks. In the Kingdom of Saudi Arabia, the majority of people have skin types IV and V. Therefore, vitiligo is linked to unfavorable effects [5].

Despite the fact that vitiligo usually does not have any symptoms and hardly ever leads to major physical illness [6], vitiligo sufferers frequently experience psychological issues, such as sadness, anxiety, and social phobia in addition to significant cosmetic disfigurement and diminished quality of life (QOL) [7]. Therefore, the QOL instrument was created to evaluate how a patient's health was affected by their illness [8]. The QOL of people with vitiligo has been linked to numerous conditions, such as the level of education, younger age group, female gender, site, and severity of the lesion [9].

A recent study done in AL Qassim, Saudi Arabia, found that the average DLQI score for all patients was 14.72 (SD ± 5.173). The study revealed that women experience more embarrassment and self-consciousness due to the disorder, leading to more significant impacts on their personal relationships, social life, and sexual activities compared to males [10]. This study is needed to assess the QOL of patients in the eastern region of Saudi Arabia because of the lack of research related to our topic, especially in the eastern region of Saudi Arabia.

## Materials and methods

Study design and settings

A cross-sectional chart review study was performed. The study was conducted on patients with vitiligo in the eastern region of Saudi Arabia. The inclusion criteria were Saudi males and females from all social classes who were older than 18 years and diagnosed with vitiligo. The exclusion criteria were Saudi adults not diagnosed with vitiligo and those younger than 18 years old. An online survey using an Arabic version of the Dermatological Index of Quality of Life (DLQI) was distributed to the targeted population.

The sample size was calculated using the Raosoft calculator (http://www.raosoft.com), considering a 95% confidence interval, 5% margin of error, and a total population of 380; the minimum required sample size was 263.

QOL measurement tool

We used the DLQI, which was created as a research instrument in 1994 by Great Britain [11]. The questionnaire has 10 inquiries that assess the patient's personal, social, sexual, household, and professional aspects. We were granted authorization to use the Arabic version only for the purpose of this investigation. The equivalent points are as follows: 0-1 point - No impact on the life of the patient; 2-5 points - Disease has an intolerable impact on the life of the patient; 6-10 points - The patient's life is moderately affected by the illness; 11-20 points - Disease has a profound impact on the patient's life; 21-30 points - The patient's life is extremely strongly affected by the illness.

Analyzes and entry method

The data were inputted into the computer using the Excel 2016 (Microsoft® Corp., Redmond, WA) application designed for the Windows operating system. The data were then uploaded to the Statistical Product and Service Solutions (SPSS, version 29.0.0; IBM SPSS Statistics for Windows, Armonk, NY) software to conduct statistical analysis.

Statistical analysis plan

A comprehensive statistical analysis was conducted on the dataset, encompassing both descriptive and inferential methodologies. Firstly, a descriptive analysis is conducted to summarize the participants' demographic characteristics, which include age, gender, and other features. This provides an overview of the study population. Subsequently, inferential analyses such as Fisher's exact tests or chi-square tests are employed to examine the associations between categorical variables. The linear regression model is used to find predictors of the severe influence of vitiligo on patients' QOL. Statistical significance is established at a p-value ≤ 0.05 and a confidence interval of 95%. All statistical analyses are executed using SPSS software.

## Results

Our study comprises 263 participants from the eastern region of Saudi Arabia; the gender distribution shows 55.1% females and 44.9% males. Regarding age, 54.4% are between 18 and 30 years old, while 45.6% are older than 31. In terms of marital status, 68.8% are married, and 31.2% are single. All participants reside in the eastern region, and the entire sample, 100%, has been diagnosed with vitiligo (Table 1).

**Table 1:** Sociodemographic parameters of participants

		Frequency (n=263)	Percent
Gender	Female	145	55.1
	Male	118	44.9
Age	18-30 Years	143	54.4
	>31 Years	120	45.6
Marital Status	Single	82	31.2
	Married	181	68.8
Live in Eastern Region	Yes	263	100.0
Diagnosed with Vitiligo	Yes	263	100.0

Table 2 shows the influence of vitiligo on the QOL among patients in the eastern region of Saudi Arabia. Regarding physical symptoms, 76.8% reported never experiencing itching, pain, or tingling, while 3.4% experienced it very much. The emotional toll of vitiligo is evident, with 56.3% feeling very much embarrassed and uncomfortable. Daily activities, such as household chores, were affected a lot by 42.6%, and clothing choices very much by 43.7%, indicating a substantial impact. Social and leisure activities were influenced a lot by 53.2%. Work or training problems were reported by 49.4%, and sporting activities impacted 30.4%. Notably, relationships were affected very much for 36.5% and sexual problems a lot for 35.7%. Treatment-related difficulties were acknowledged a lot by 51.3% of respondents.

**Table 2:** Questionnaire assessing the quality of life (QOL) of vitiligo patients

Questions		Response			
		Never	Little	A lot	Very Much
Over the past year, how often have you experienced itching, pain, or tingling in your skin?	N	202	46	6	9
	%	76.8	17.5	2.3	3.4
Over the past year, to what extent have you felt embarrassed and uncomfortable because of your vitiligo?	N	28	40	47	148
	%	10.6	15.2	17.9	56.3
Over the past year, how much has vitiligo affected your household, outdoor chores, or shopping?	N	41	51	112	59
	%	15.6	19.4	42.6	22.4
Over the past year, to what extent has vitiligo affected your choice of clothing?	N	28	37	83	115
	%	10.6	14.1	31.6	43.7
During the past year, to what extent has vitiligo affected your social activities and leisure time?	N	32	47	140	44
	%	12.2	17.9	53.2	16.7
During the past year, to what extent did vitiligo affect your sporting activities?	N	80	80	66	37
	%	30.4	30.4	25.1	14.1
To what extent has vitiligo caused problems for you in your work or training?	N	36	97	130	0
	%	13.7	36.9	49.4	0.0
Over the past year, how bad have your problems been caused by vitiligo with your partner, close friends, or relatives?	N	41	51	75	96
	%	15.6	19.4	28.5	36.5
Over the past year, how bad has vitiligo affected your sexual problems?	N	51	62	94	56
	%	19.4	23.6	35.7	21.3
To what extent has treatment for vitiligo caused you difficulties?	N	26	44	135	58
	%	9.9	16.7	51.3	22.1

**Figure 1:**
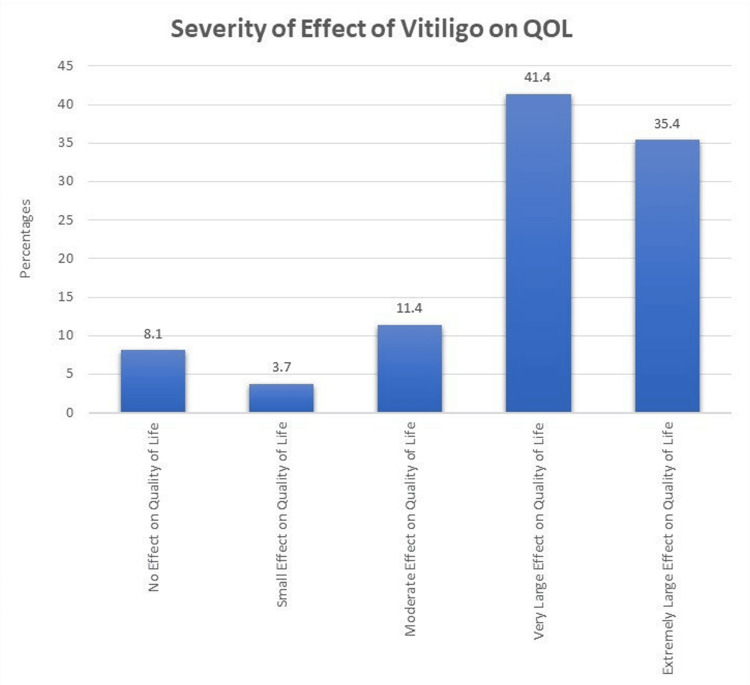
Different categories of severity on QOL in vitiligo patients

Figure 1 categorizes the severity of the influence on the QOL among vitiligo patients. Notably, 8.1% report no effect on their QOL, 3.7% experience a small effect, 11.4% indicate a moderate effect, and a significant portion, 41.4%, faces a very large impact. Furthermore, 35.4% express an extremely greater influence on their QOL.

Table 3 shows the results of a linear regression model assessing predictors of the severity of QOL in vitiligo patients. Gender (male) has a negative coefficient of -2.372 (p = 0.008), suggesting that the male gender significantly experiences less impact of vitiligo on QOL severity. Age and marital status, however, show non-significant associations with coefficients of -0.647 (p = 0.485) and 0.053 (p = 0.958), respectively. The standardized coefficients (beta) provide a measure of the relative importance of predictors, with gender having the most substantial impact.

**Table 3:** Predictors of severity of QOL in vitiligo patients (linear regression mode) *Male gender significantly experiences less impact of vitiligo on QOL severity

	Unstandardized Coefficients		Standardized Coefficients	Sig.	95% CI	
	B	Std. Error	Beta		Lower Bound	Upper Bound
(Constant)	18.522	2.450		0.000	13.698	23.345
Gender (Male)	-2.372	0.883	-0.164	0.008*	-4.111	-0.632
Age	-0.647	0.926	-0.045	0.485	-2.470	1.176
Marital Status	0.053	0.995	0.003	0.958	-1.907	2.013

Table 4 shows the association between the severity categories of vitiligo and different parameters, namely, gender, age, and marital status. A significant association is observed between gender and severity (p < 0.001), indicating that a higher proportion (n = 70, 48.3%) of females experience a very large effect on their QOL compared to males (n = 39, 33.1%). Conversely, age and marital status do not show significant associations with severity, as indicated by p-values of 0.255 and 0.678, respectively.

**Table 4:** Association between different categories of severity among different parameters (Fisher’s exact test) *Significant association observed between gender and severity

		Gender			Age			Married		
		Female	Male	p-value	18-30 Years	>31 Years	p-value	Single	Married	p-value
No Effect	N	1	20	<0.001*	9	12	0.255	6	15	0.678
	%	0.7%	16.9%		6.3%	10.0%		7.3%	8.3%	
Small Effect	N	5	5		3	7		3	7	
	%	3.4%	4.2%		2.1%	5.8%		3.7%	3.9%	
Moderate Effect	N	19	11		20	10		13	17	
	%	13.1%	9.3%		14.0%	8.3%		15.9%	9.4%	
Very Large Effect	N	70	39		59	50		32	77	
	%	48.3%	33.1%		41.3%	41.7%		39.0%	42.5%	
Extremely Large Effect	N	50	43		52	41		28	65	
	%	34.5%	36.4%		36.4%	34.2%		34.1%	35.9%	

## Discussion

Vitiligo is a condition where melanocytes, which are responsible for producing pigment, are lost, resulting in depigmented areas on the skin and mucous membranes. This condition is typically accompanied by leukotrichia [12]. Vitiligo exhibits a higher prevalence in females. Similarly, Patil et al. showed that older males had a lower tendency to report vitiligo in the clinic as compared to older females [13]. This progressive hypo-melanosis primarily impacts facial and extremity regions. Al-Smadi et al. [14] demonstrate that the areas often exhibiting hyperpigmentation are commonly impacted by vitiligo, including the inguinal/anogenital regions, sacrum, umbilicus, axillae, nipples, the dorsal surfaces of the hands, and the periorificial region of the face [14]. Etiology involves genetics, oxidative stress, autoimmunity, and more [15]. In Saudi Arabia, where vitiligo affects skin types IV and V, it has significant psychosocial impacts. Despite being symptomless, it induces psychological issues. Recent Saudi research indicates greater impairment in women. Our study aimed to comprehensively assess the impact of vitiligo on QOL and identify potential predictors of severity. 

Notably, the demographic parameters of our sample correspond to the distribution reported in other regional studies. The predominance of females (55.1%) is consistent with the generally higher prevalence of vitiligo among women. An investigation conducted by Zhang et al. revealed a significantly high occurrence of vitiligo in African regions and among female patients [16]. The age distribution, with a significant proportion (54.4%) aged 18-30 years, corresponds to the age range commonly affected by this condition. Mahajan et al. showed that the mean age at the onset of vitiligo was 20.5 years [17]. Marital status indicates a higher prevalence among married individuals (68.8%), possibly linked to the psychosocial implications of the condition on relationships and family dynamics. Prior research indicates that vitiligo has been seen to have a detrimental effect on marital relationships, including cases where the appearance of skin depigmentation has led to divorce [18,19].

Regarding the multifaceted impact of vitiligo on various aspects of QOL, the high percentage (76.8%) reporting no physical symptoms suggests a subgroup with a relatively milder form of the condition. However, the emotional toll is pronounced, with over half experiencing significant embarrassment and discomfort. Ezzedine et al. showed that vitiligo has been associated with anxiety and depression [20]. Daily activities and clothing choices are substantially affected, underscoring the challenges individuals face in navigating routine tasks and expressing themselves. The observed impact on social, leisure, and sporting activities emphasizes the broader societal implications of vitiligo. Work or training problems reported by nearly half of the participants highlight potential challenges in professional and educational domains. The significant impact on relationships (36.5%) and sexual problems (35.7%) accentuates the need for a holistic approach to address the psychosocial dimensions of vitiligo. Maamri et al. showed that the severity of vitiligo was negatively correlated with sexual satisfaction [21].

Notably, the severity of QOL impact revealed a considerable proportion (41.4%) facing a very large effect and 35.4% expressing an extremely large effect. These findings resonate with the existing literature, where vitiligo is consistently associated with a substantial negative impact on QOL.

Notably, the linear regression model provides insights into predictors of QOL severity. Gender emerges as a significant predictor, with males experiencing less impact, aligning with some existing studies. According to Samela et al., women with vitiligo are more likely to have a greater risk of experiencing poorer mental health and a worse QOL, including a wider spectrum of psychopathology symptoms, compared to males [22]. The non-significant associations with age and marital status highlight the complex and multifactorial nature of QOL in vitiligo patients.

Notably, our study reveals associations between severity categories and demographic factors. A significant association between gender and severity further emphasizes the gender-specific impact of vitiligo on QOL. Interestingly, age and marital status do not exhibit significant associations, indicating that the severity of vitiligo's impact may not be influenced by these demographic factors.

Comparing our findings with existing literature, our study reinforces the widely documented psychosocial impact of vitiligo on various aspects of QOL. The observed gender-specific differences align with studies that suggest variations in the emotional and social implications of vitiligo between males and females. The non-significant associations with age and marital status, however, diverge from some previous reports, highlighting the need for further exploration of these factors in diverse cultural contexts.

Limitations

Several study limitations include potential biases due to cultural variations, limited generalizability beyond the eastern region, and reliance on self-reported data for psychological aspects.

## Conclusions

Our study contributes valuable insights into the specificities of vitiligo's impact on QOL in the eastern region of Saudi Arabia. While affirming the psychosocial challenges documented in global literature, our findings also emphasize the importance of considering gender dynamics in understanding the nuanced experiences of individuals with vitiligo. These insights can inform tailored interventions aimed at enhancing the QOL of vitiligo patients in this region.
